# BBNet: A Novel Convolutional Neural Network Structure in Edge-Cloud Collaborative Inference

**DOI:** 10.3390/s21134494

**Published:** 2021-06-30

**Authors:** Hongbo Zhou, Weiwei Zhang, Chengwei Wang, Xin Ma, Haoran Yu

**Affiliations:** 1 College of Engineering, Huaqiao University, Quanzhou 362021, China; 19014084013@stu.hqu.edu.cn (H.Z.); 1895121032@stu.hqu.edu.cn (C.W.); xinma@stu.hqu.edu.cn (X.M.); 19013082039@stu.hqu.edu.cn (H.Y.); 2 Fujian Provincial Academic Engineering Research Centre in Industrial Intellectual Techniques and Systems, Quanzhou 362021, China

**Keywords:** collaborative intelligence, deep learning, model compression, feature compression, cloud computing

## Abstract

Edge-cloud collaborative inference can significantly reduce the delay of a deep neural network (DNN) by dividing the network between mobile edge and cloud. However, the in-layer data size of DNN is usually larger than the original data, so the communication time to send intermediate data to the cloud will also increase end-to-end latency. To cope with these challenges, this paper proposes a novel convolutional neural network structure—BBNet—that accelerates collaborative inference from two levels: (1) through channel-pruning: reducing the number of calculations and parameters of the original network; (2) through compressing the feature map at the split point to further reduce the size of the data transmitted. In addition, This paper implemented the BBNet structure based on NVIDIA Nano and the server. Compared with the original network, BBNet’s FLOPs and parameter achieve up to 5.67× and 11.57× on the compression rate, respectively. In the best case, the feature compression layer can reach a bit-compression rate of 512×. Compared with the better bandwidth conditions, BBNet has a more obvious inference delay when the network conditions are poor. For example, when the upload bandwidth is only 20 kb/s, the end-to-end latency of BBNet is increased by 38.89× compared with the cloud-only approach.

## 1. Introduction

In recent years, deep learning has achieved awesome performance in various smart-application scenarios [1,2]. At present, the most common DNN deployment methods are mobile-only and cloud-only [3]. Mobile-only deploys the DNN model on an edge device. This deployment method can only handle simple inference tasks in edge devices. Regarding cloud-only, it deploys the DNN model in the cloud, sends the original data directly to the cloud, and finally returns the inference result. In this way, the original data are transmitted in the channel, which is not only a threat to sensitive data but also increases communication delay. Kang et al. [4] proposed a new method of deep neural network partition deployment to achieve the effect of joint inference on the edge device and the cloud. This method is called collaborative intelligence. By this means, the edge device deploys the early layer of the neural network and uploads the intermediate feature data to the cloud to execute the remaining network layer.

In the status quo approaches, there are two methods for edge-cloud collaborative inference-based convolutional neural networks (CNN). On the one hand, the initial method is not to change the structure of the CNN [4,5,6]. It is to directly divide the CNN from a certain layer in the middle, but because of the in-layer data amplification [7], the split point will always be in the later layer of CNN. Eshratifar et al. [8] pointed out that in the collaborative method, more than 75% of the total energy and delay costs are caused by communication. On the other hand, it is to change the deep network structure [9,10]. In BottleNet [10], the author introduces a bottleneck layer to reduce the feature data size transmitted by the middle layer and speed up the CNN inference process. Chen [9] proposed an early exit mechanism to terminate the calculation on the edge device at an appropriate point to promise application latency requirement. These works have made a lot of progress in collaborative inference, and the choice of the best split point has always been a critical issue for this mechanism. However, the choice of the best split point is usually restricted by the model itself. In addition, the trade-off between loss of accuracy and model size and speeding up inference should be considered.

**Figure 1:**
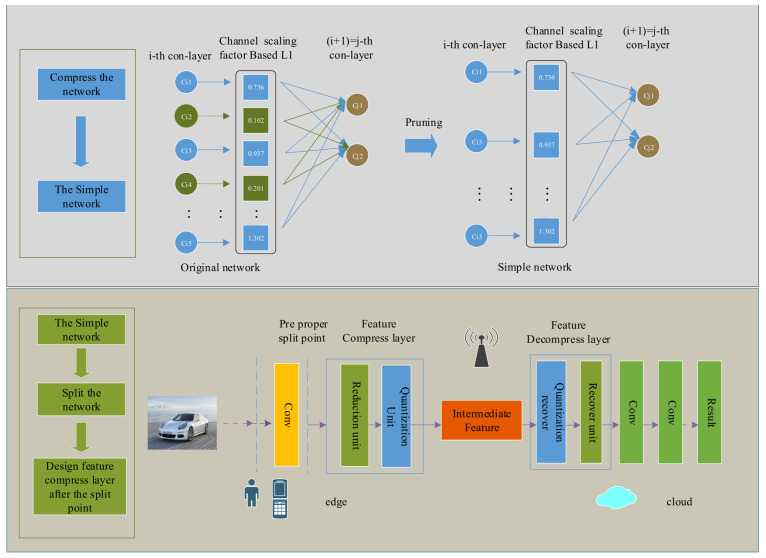
The framework of BBNet (**1**) First step is to obtain a simple network through channel-pruning. Channel-pruning associates the scaling factor on the BN layer with the channels in the convolutional layer, and then deletes the unimportant channels through sparse training. (**2**) Based on the simplified network, the feature compression layer is placed at different segmentation points to further reduce the transmission feature size. The feature compression layer is composed of a learnable feature reduction unit, which restores its lost accuracy through precision perception training.

Therefore, to address these problems, we propose a novel CNN structure in edge-cloud collaborative inference—BBNet. As shown in Figure 1, The structure of BBNet is deeply decoupled from the CNN as follows two aspects: first, it compresses the complete network, to reduce the overall size of the model and the amount of calculation. When designing the network structure, we reduced the number of channels of the overall network proportionally for different classification tasks. In addition, it then added a scaling factor based on L1 regularization to all BN layers. Each scaling factor corresponds to a specific convolutional channel. By setting the threshold, we can identify less important channels (or neurons). Liu [11] pointed out that pruning unimportant channels may temporarily reduce the performance of the model, but by global fine-tuning of the pruned model will obtain a model with higher generalization ability, fewer parameters, and lower runtime memory. Second, based on obtaining a simplified network in the first step, we partition the convolutional neural network. Part of the CNN is calculated on the edge device, and the other is sent to the cloud. Since the intermediate features of the deep model have lower entropy compared with the original model [10], inspired by this, we introduced a feature compression layer after the optimal split point to reduce the size of the transmitted feature tensor and reduce the cost of communication.

The BBNet collaborative inference structure can be applied to general supervision tasks. This structure can be roughly understood as treating the channel in the CNN as the latitude of the model, and treating each pre-partition point as the longitude of the model. Then BBNet optimizes the model from the latitude and longitude of the model, therefore speeding up the edge-cloud collaborative inference.

Our main contributions are as follows:

- We propose a novel convolutional neural network structure in edge-cloud collaborative inference which economizes end-to-end latency through accelerated inference from two directions.
- Improved model compression, which can reduce the number of model calculations and parameters while the accuracy loss is small.
- We designed the feature compression layer, which can achieve the highest bit-compression rate within the defined accuracy loss range.
- Finally, the BBNet prototype was implemented on NIVIDIA Nano and the server. The evaluation results based on different static network bandwidths demonstrate the effectiveness of the proposed BBNet framework.

The remainder of the paper is structured as follows: Section 2 provides backgrounds and related technical information for BBNet. Section 3 provides a more detailed design of BBNet. In Section 4, we give the test environment on the actual hardware and compare the experimental results of different approaches. Section 5 gives the summary of our paper and proposals for future works.

## 2. Related Work

Based on the related work, we proposed the structure of BBNet shown in Figure 1, which combines three technologies, namely model compression, DNN model partition, and feature compression. In the next few subsections, we will introduce the relevant work from these three aspects. Due to the limited computing resources of edge devices, model compression techniques are used to reduce the overall model size and speed up inference when designing the BBNet structure. This step is called the latitude from the model to reduce the end-to-end latency. In addition, affected by the in-layer data amplification and the redundancy in intermediate features, BBNet also compresses the features after the split points, which is called for by the longitude of the model to reduce the end-to-end latency. More specific details are introduced in Section 3.

### 2.1. Model Compression

The current mainstream model compression methods include parameter quantification [12,13], network-pruning [11,14,15], and knowledge distillation [16]. DNN parameters mostly use floating-point numbers. Parameter quantization maps floating-point numbers to a certain range so that the size of the feature map can be reduced [7], and the computationally expensive floating-point operations are avoided. Network-pruning is roughly divided into weight-pruning and channel-pruning. The point of network-pruning is to delete the least important channels or weights. The point of weight-pruning is to remove individual neurons in the filter or connections between neurons across different layers [17]. However, due to the irregular network structure after pruning, this strategy requires special software and hardware support. To further improve the compression ratio, Deep Feature Compression [14] combines pruning, weight-sharing, and Huffman coding to compress DNN. The point of channel-pruning is usually to remove unimportant channels between layers. In Network-Slimming [11], the author proposed a method of channel-level sparsity in the network which way is simple but effective. This method does not require special software/hardware accelerators. In the channel-pruning method, we were inspired by this. Knowledge distillation is mainly aimed at training a student network from the output prediction data of a teacher network [16], to reduce the model size and computing resources while maintaining the same accuracy as the teacher network.

Model compression provides an elementary way to reduce model size and accelerating the execution. Considering the redundancy in the model channel, the structural design of BBNet is based on one form of model compression, which called channel-pruning. Then, we introduce two approaches to further improve the speed for more effective collaborative inferences. These two approaches are introduced in the following two subsections.

### 2.2. DNN Model Partition

Many works offload the DNN inference task from local to the cloud [4,8,18,19,20] to make full use of the computing resources between edge and cloud. In Neurosurgeon [4], Kang et al propose a partially offloaded scheme. It consists of two parts, first determining a partition point in the DNN structure, and keeping the front layer in the local device, then offloading the remaining layer to the cloud for collaborative inference. DDNN [18] uses a similar principle and map sections of a DNN onto a distributed computing hierarchy. DDNN can accommodate the DNN inference in the cloud, while also allowing fast, localized inference using the shallow part of the neural network at the edge and terminal devices. JointDNN [8] uses a graph-based method to explain the general architecture of DNN layers and formulate the computing scheduling problem in the mobile cloud computing environment as the shortest path problem and ILP problem. Moreover, JointDNN also uses lossless encoding (PNG compression) to reduce the amount of intermediate data transmission. Their works illustrate that the DNN model partition can achieve low-latency inference with a small loss of accuracy.

Recently, more and more work has adopted DNN model partition in actual intelligent applications [21,22,23]. FedHmome [22] is a joint learning framework for home health detection based on edge-cloud, which learns a shared global model in the cloud from multiple families at the edge of the network. The work of Wang et al. [23] proposed a dynamic resource allocation scheme to select the best division point of DNN inference tasks in the intelligent application of vehicles. These works indicate that DNN model partition has become an effective model inference method.

### 2.3. Feature Compression

Although DNN model partition can significantly reduce the end-to-end delay, the location of the split point is still restricted by the structure of the model itself. Many works have further processed the feature data in the DNN [24]. For example, in JALAD [7], Li et al. used quantization and Huffman coding to compress in-layer feature maps to reduce the data size. In semantic image compression, the work in [25] achieves the purpose of compression by encoding deep features and then reconstructing the input image from them. In [5], the author proposed a deep feature compression structure, which consists of a pair of encoders and decoders and can be applied to the environment of edge-cloud collaborative inference. However, the above work is all processed on the output feature data of the original DNN, and the structure of the DNN is not changed. In BottleNet [10], the author proposed a bottleneck unit, which is composed of a learnable lossy compressor and uses compressed sensing training to reduce the accuracy loss caused by lossy compression. Follow-up work also has done research on multi-task learning [26], considering the sparsity in the feature maps which motivate the emergence of feature compression layer after split point.

Our work belongs to the category of edge-cloud collaboration, which integrates channel-pruning, model partition and feature compression technologies to accelerate DNN inference from the two direction of mode latitude and longitude. In addition to the novel design combining these technologies, BBNet also considers adaptively finding the best split point under different bandwidth conditions to achieve the best inference effect.

## 3. Proposed Method

This section first describes a channel-pruning scheme to reduce the size of the CNN model (FLOPs and parameter), and then introduces the selection of partition points and the detailed design of the feature compression layer. Our optimization objective is to accelerate the speed of collaborative inference within the scope of a small loss of accuracy.

### 3.1. Channel-Pruning

Due to channel-level sparsity, this does not require special libraries to obtain the benefits, and can produce considerable model-size compression and accelerated inference [11]. BBNet chooses the channel-pruning method to remove unimportant channels. The specific details are presented next.

#### 3.1.1. Scaling Factor and Sparsity

Motivated by the fact that the in-layer feature map exhibits good sparsity and the DNN model has a high generalization ability, this paper first selectively reduces the number of channels of the CNN model before training according to different classification tasks. Second, this paper uses a scale factor γ label for each channel and multiplies it by the output of that channel. Then, the joint weights and these scale factors are used for training, and L1 regularization is used to restrict the model, and the model is processed in the direction of sparseness. Finally, we trim the channels below the threshold and fine-tune the network. The overall training loss target is as follows:

(1) $$L=\sum_{\left(x,y\right)}l\left(f\left(x,w\right),y\right)+\lambda \sum_{\gamma \in \tau }g(\gamma )$$

Among them, *L* is the overall loss function. $\sum_{\left(x,y\right)}l(f(x,w),y)$ is the loss function under normal training conditions, where (*x*,*y*) denotes the train input and target, and *w* denotes the weight.

$\lambda \sum_{\gamma \in \tau }g(\gamma )\tau$ is the loss function caused by sparsity, γ denotes a scaling factor for each channel, and λ is used to balance two terms. In addition, the state space of γ is represented by τ.

When $g\left(\gamma \right)=\left|\gamma \right|$, it is expressed as using L1 regularization to restrict the scale factor to achieve sparsity. We use the sub-gradient descent method for the non-smooth L1 penalty term. Channel-pruning is supposed to delete all the inputs and outputs of the channel, and then it will create a simple network. The scale factor and weight are optimized at the same time during training to identify less important channels without affecting the generalization ability of the network model.

#### 3.1.2. The Scaling Factor in BN Layer

The BN layer is adopted in most modern CNNs as a standard method to achieve fast convergence and better generalization performance. We use mini-batch statistics in the BN layer to standardize internal activations to incorporate channel-wise scaling factors. The conversion formula performed in the BN layer is as follows, where $Z_{in}$ and $Z_{out}$ represent the input and output of the BN layer.

(2) $$Z_{out}=\alpha \hat{z}+\beta$$

Among them, α and β are trainable affine transformation parameters (scale and shift), which provide the possibility to transform the normalized activation linearly to any scale. We directly use α as the scaling factor, so that it does not bring extra overhead to the network. $\hat{z}$ is the value to be input to be standardized, and the specific formula is as follows:

(3) $$\hat{z}=\frac{z_{in}-\mu_{\beta }}{\sqrt{{\delta_{\beta }}^{2}+\varepsilon }}$$

where $\mu_{\beta }$ and $\delta_{\beta }$ are the average and the standard deviation value of input activations over β, and ε is the balance conversion factor.

#### 3.1.3. Channel-Pruning and Global Fine-Tuning

We can create a model with many scaling factors close to zero after the regularization training induced by the previous channel-level sparseness. Then we sort these scaling factors, and define a global threshold for this purpose, which is a certain percentage of all scale factors. In the experiment, we chose a threshold of 40%, i.e., to trim 40% of the channels with a smaller scaling factor. Through channel-pruning, a smaller model structure can be obtained. Pruning the trained model may result in a decrease in the accuracy of the model, but then through fine-tuning the model globally can make up for the loss of accuracy.

### 3.2. Feature Compression Layer

**Figure 2:**
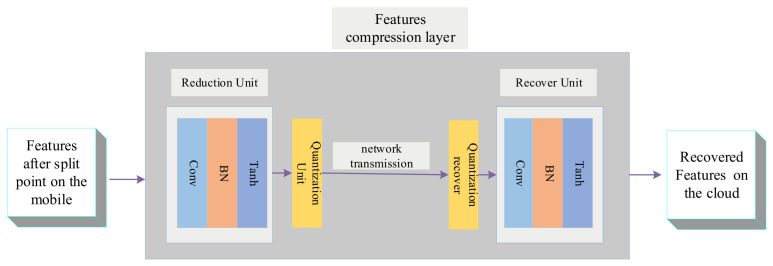
Feature compression layer design, which consists of a pair of learnable feature reduction units.

Although channel-pruning can accelerate the speed in CNN inference and its capability, from another angle, this section introduces a detailed design about feature compression to accelerate collaborative inference further. As shown in Figure 2, the feature compression layer is composed of a learnable micro-network and a quantization unit, which is responsible for learning the dense representation of intermediate features to reduce the data size in the layer. Through accuracy-aware training, we can keep the accuracy loss within reasonable constraints and maximize the data compression rate. Next, we will explain details about the feature compression layer.

#### 3.2.1. Reduction Unit and Recover Unit

The learnable micro-network is composed of a feature reduction unit and feature recovery unit. Each unit corresponds to a convolutional layer, batch normalization layer, and activation layer. The feature reduction unit is implemented in two dimensions: space reduction and channel reduction. In addition, the recovery unit adopts a similar principle. More specifically, different size convolution filters control the output of the channel, and the convolution kernel controls the spatial-dimension size of the feature. The intermediate feature tensor before compression layer can be expressed as $\left(batch\_size,c,w,h\right)$ and after compression layer is expressed as $\left(batch\_size,c^{\prime },w^{\prime },h^{\prime }\right)$. To compress the *c* channel to the $c^{\prime }$, and change spatial dimension to $h^{\prime }$ and $w^{\prime }$, the filter size is set to $c^{\prime }$ and the convolution kernel step is set to $\left[\frac{w}{w^{\prime }}\right]$ and $\left[\frac{h}{h^{\prime }}\right]$, respectively.

In the experiment, the same spatial-dimension scaling factor size is used, i.e.,  $\left[\frac{h}{h^{\prime }}\right]$. It is worth noting that to add nonlinear features after the compression layer, we added a batch normalization layer and an activation layer after the convolution layer. We use the Tanh function, because the output value is limited to the range of $\left[-1,1\right]$, which is more conducive to further quantifying the data in network communication.

#### 3.2.2. Quantization Unit

At present, most DNN parameters are represented by 32-bit floating-point numbers, which is a waste of communication resources in network transmission. In addition, recently there has been proof of work that only using 4-bit to represent DNN parameters. Its impact on accuracy will not exceed 1% [27]. Therefore, 8-bit is selected in the experiment to quantify the intermediate eigenvalues. The quantitative formula is as follows:

(4) $$V^{\prime }=round\left[\left(V+1\right)\cdot \left(2^{n}-1\right)\right],V\in \left(-1,1\right)$$

where $V\in R^{C\times W\times H}$ is the feature tensor after the reduction unit, and *C*, *W*, and *H* correspond to the number of channels, width, and height of the tensor, respectively. The difference between this method and the quantization method in [5] is that in [5] they need to upload the max(V) and min(V) in the tensor to the cloud, but we do not need it. This is because we chose Tanh as activation function. Next, to save the transmission bandwidth, we use the small byte to represent the quantized value. The quantization-recover unit is the inverse process of the above formula, but this process is done in the cloud.

#### 3.2.3. Accuracy-Aware Training

Inserting the feature compression layer after the simple network model will inevitably lower the generalization ability of CNN. To solve this problem, we will adopt the accuracy-aware training method. First, to save training time, we freeze all layers except the feature compression layer [28]. Second, we keep the accuracy loss within the constraint range, and obtain the maximum compression ratio of each partition point by iterative training. It is noteworthy that after the Accuracy-Aware Training, the compression ratio of each split point will be determined.

### 3.3. Formulation of Partition Point

Based on the learnable feature compression layer, we propose a deep structure partition strategy to reduce the end-to-end inference latency. Before obtaining the best split point, the end-to-end latency of processing the input data must be measured. The end-to-end latency consists of three parts, the edge device processing latency ${TM}_{i}$, the transmission latency ${TU}_{i}$, and the cloud processing latency ${TC}_{i}$. We use $i\in \left[1,2,\ldots ,N\right]$ to denote the number of layers of the deep neural network, and *N* to denote the maximum index of the DNN layer. The proposed algorithm mainly includes three stages: (1) training; (2) analysis; (3) selection. Algorithm 1 gives a detailed description. For a simplified network model, which has *L* different positions, we place the feature compression layer after the pre-partition point of the network model. In the experiment, we chose the ResNet structure with branch structure. In addition, driven by accuracy-aware training, we obtain the structure with the best compression rate. For the solution of *L* different split points, according to optimization goal, we choose the minimum end-to-end latency as our best partition strategy. *Z* is the end-to-end latency, and $x_{i}$ is a binary variable, indicating whether to choose the i-th layer as the split point. The overall optimization goal is as follows:

(5) $$\begin{array}{c} \begin{array}{cc} \; & \min\;Z=\sum_{i=1}^{N}TM_{i}\cdot x_{i}+\sum_{i=1}^{N}TU_{i}\cdot x_{i}+\sum_{i=1}^{N}TC_{i}\cdot x_{i}\\ \; & s.t\;\;\sum_{i=1}^{N}x_{i}=1\\ \; & \mathrm{vars}\;x_{i}\in \left\{0,1\right\} \end{array} \end{array}$$

In this way, we programmed finding best split point *i*-based end-to-end latency. We further study the time complexity to solve this problem.

From Algorithm 1, we can receive a result that the time complexity is $O\left(N*{C^{\prime }}_{\max}*S_{\max}\right)$. The time complexity of the algorithm depends on the size of *N*, ${C^{\prime }}_{\max}$ and $S_{\max}$. However, when there is a large search space, we adopt an early exit mechanism which is based on accuracy. In the initial stage, an accuracy threshold range will be given. If the training accuracy of model is already within the threshold range, the algorithm will jump out of the loop and continue to execute the next layer.
**Algorithm 1** The partition algorithm.**Input:***N*: The numbers of layers in the DNN*L*: The numbers of partitioning points in the DNN$K_{mobile}$: current load level of mobile$K_{cloud}$: current load level of cloud$t_{mobile}\left(j,K_{mobile}\right)$: The latency in mobile with j partitioning point and $K_{mobile}$ load level$t_{cloud}\left(j,K_{cloud}\right)$: The latency in mobile with j partitioning point and $K_{cloud}$ load level$S_{\max}$: The max of spatial scaling factor${C^{\prime }}_{\max}$: The max of channel number${model}_{\mathrm{compact}}$: The compact model after Channel-pruningNB: The bandwidth of wireless networkacc: The accuracy of training model**Output:**The best partitioned mode**Variables:**$\left\{D_{j}\|j=1,2,\ldots ,M\right\}$: The compressed data size in each of M splitting point//train phase1:**for** i = 1; i <= N; i++ **do**2:    **for** C′ = 1; C′ <= ${C\prime }_{\max}$; C′++ **do**3:        **for** S = 1; S <= $S_{\max}$; S++ **do**4:           Place feature compression layer-based ${\mathrm{model}}_{\mathrm{compact}}$5:           Train6:           **if** acc >= target accuracy **then**7:               Save8:               Break()9:           **end if**10:        **end for**11:    **end for**12:**end for**//implementation phase13:**for** i = 1; i <= N; i++ **do**14:    ${\mathrm{TM}}_{i}$ = $t_{mobile}\left(j,K_{mobile}\right)$15:    ${\mathrm{TU}}_{i}$ = $D_{j}/\mathrm{NB}$16:    ${\mathrm{TC}}_{i}$ = $t_{cloud}\left(j,K_{cloud}\right)$17:**end for**//select phase18:**if**OptTarget is min latency **then**19:    return $argmin{}_{j=1\ldots M}\;\left(TM_{i}+TU_{i}+TC_{i}\right)$20:**end if**

## 4. Evaluation

### 4.1. Experiment Setup

#### 4.1.1. Edge and Cloud Settings

We implemented a BBNet prototype based on NVIDIA JETSON NANO and a PC to verify the feasibility and efficiency of BBNet. The detailed hardware configuration is shown in Table 1 and Table 2. In addition, we use WonderShaper tool [29] to control the network bandwidth, and define the accuracy loss boundary. It is worth noting that in the actual time latency measurement, there are sometimes slight differences of a few milliseconds under the same conditions. We take the average value after multiple measurements to ensure the accuracy of the data and set the batch size to 100, which means that the end-to-end delay is the result of processing 100 pictures at a time.

#### 4.1.2. Model Selection and Pre-Partition Point Selection

**Figure 3:**
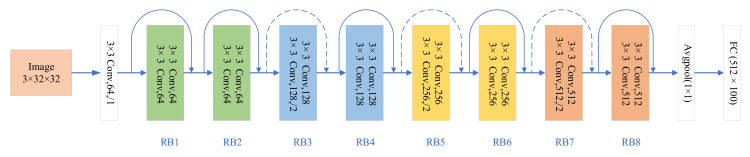
ResNet-18 architecture and different residual blocks.

**Figure 4:**
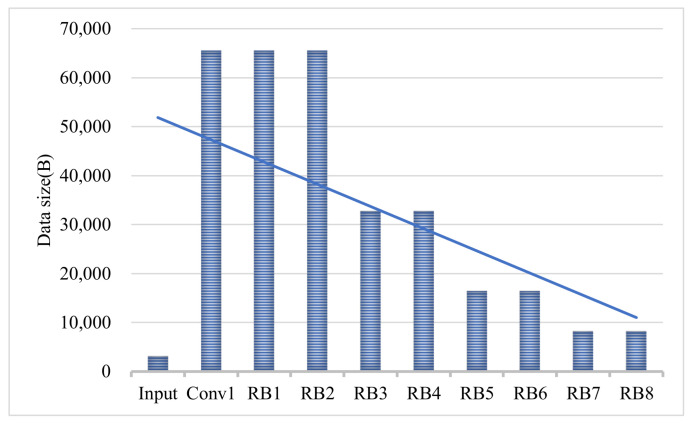
Original image size and the size of different layers in ResNet18.

The ResNet18 [30] model was selected as backbone network, and the CNN structure was built using the Pytorch [31] framework. We consider the first convolutional layer as the first pre-partition point. For the subsequent RB (residual block) layer, we use the number of channels as a benchmark, and the same number of channels is regarded as a pre-split point. As shown in Figure 3, it is the structure of RestNet18 which contains five different split point (block 1–5). In contrast to the original model structure, we use a 3×3 convolution kernel size instead of 7×7 for the size of the convolution kernel in the first convolution layer. Although this will increase the computational complexity of the model, its classification accuracy will increase a lot, so we use the modified ResNet18 as our benchmark model. As shown in Figure 4, the size of the intermediate feature tensor of ResNet18 is always larger than the size of the model input image. Therefore, it is difficult to accelerate collaboration inference only by directly dividing the network in the mobile edge and the cloud.

#### 4.1.3. Dataset

We choose CIFAR10 as the training set and test set. CIFAR10 is composed of natural images with a resolution of 32×32 and contains a total of 60,000 pictures, comprising 50,000 in the training set and 10,000 in the test set. CIFAR10 is composed of 10 categories. We use a standard data enhancement scheme [30,32,33] to rotate, shift and mirror the image. In addition, the input data are normalized using the channel average and standard deviation.

### 4.2. Reduce Model Size

The first step is to delete the unimportant channels through the pruning strategy from a channel perspective. We call this model latitude, reducing the number of computing resources required, and accelerating edge-cloud collaborative inference at the same time. Table 3 shows the diversification of accuracy, parameters and FLOPs in different ResNet18 model. BBNet achieves a significant savings in both parameters and FLOPs within small accuracy loss compared with baseline. More specifically, the parameter saving is up to 11.5× and the FLOPs reductions are around 5.6× compare between baseline and Fined-Tuned. The setting of baseline is the ResNet18 model with the size of the convolution kernel adjusted. For Cifar10, we reduced the number of channels proportionally when creating the model, which has little effect on the classification results. The channel of ResNet18 has been reduced with a pruning rate of 40%, and its accuracy has dropped a lot, but after global fine-tuning, its accuracy can be restored to a good level. The accuracy after fine-tuning is 0.53% lower than that of the baseline because the method of pruning optimization is based on the reduce channel model.

**Figure 5:**
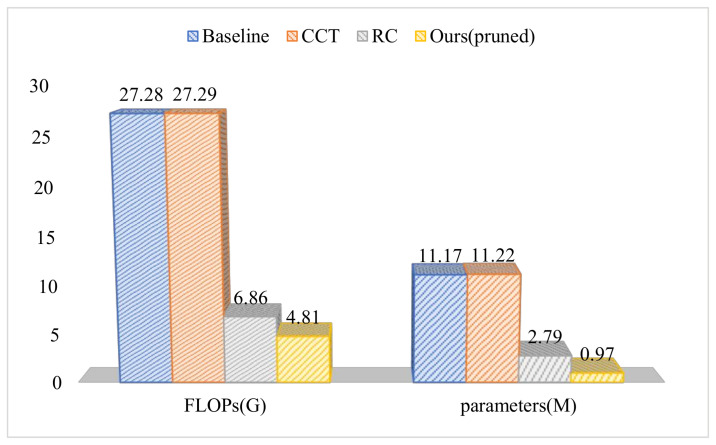
Comparison of FLOPs and parameter in different ResNet18 models.

We also compared the structure of ResNet18 in other work, which is represented by CCT [34] in Figure 5. In addition, RC is an abbreviation of reduce channel. Through the comparison of different models, BBNet has significant savings in computational cost. Therefore, our method can achieve fast inference on resource-constrained devices.

### 4.3. Bit-Compression Rate of Different Split Points

In the structure of edge-cloud collaborative inference, the end-to-end latency overhead mainly includes the calculation latency on the device and communication latency. In the second stage of the work, we put the feature compression layer at different split points. Then use accuracy-aware training to select different bit-compression rates for different partition points. We call it the longitude of the model, reducing the size of the transmitted feature data, and speeding up the edge-cloud collaborative inference. This method will achieve the maximum bit-compression rate within the threshold range of the accuracy loss. In the experiment, we set the accuracy loss threshold to 2.5% and epoch size set to 160.

**Figure 6:**
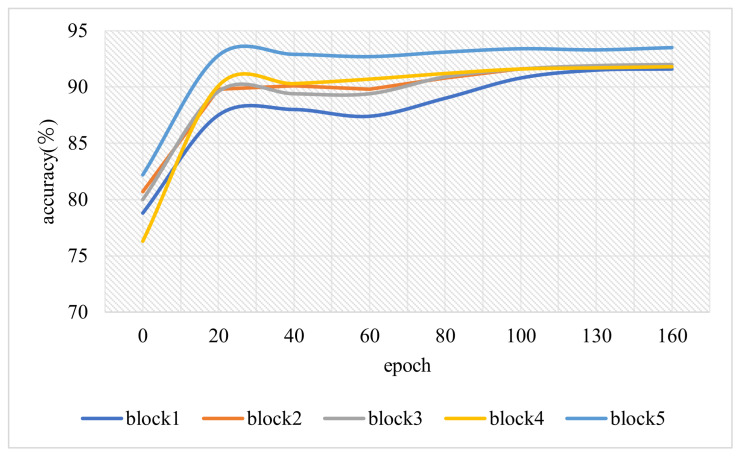
Variation of accuracy in different block of best bit-compression in BBNet.

Figure 6 plots the accuracy changes of different split points under accuracy-aware training. Due to the iterative search for different bit-compression ratios, only the curve of the maximum bit-compression ratio is shown in the figure. In addition, the experiment is based on a simple network, and other layers are frozen to save training time. Therefore, it can be observed that the model can reach high accuracy from the beginning of training.

**Figure 7:**
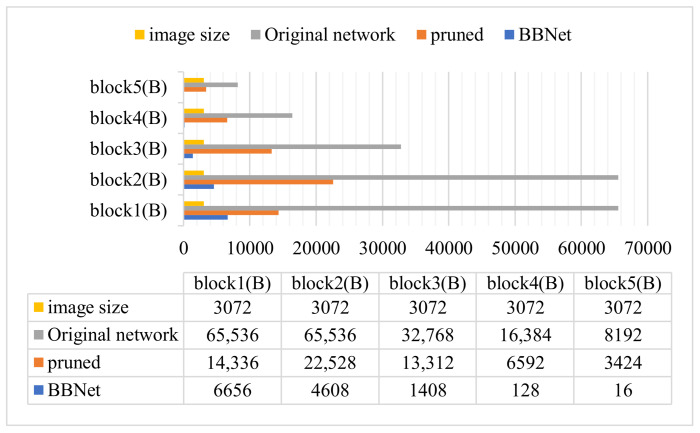
The output data size after each pre-partition point (block 1–5) in different ResNet18 structures.

Next, Figure 7 shows the original model, channel-pruning, and the bit relationship between different split points of BBNet and the original picture size. It can be clearly seen that the intermediate output data size of the original model is much larger than the original picture, which is caused by the effect of the intermediate layer data amplification [7]. In addition, BBNet has achieved a good bit-compression rate at every split point. Compared with the original model, BBNet can achieve a compression rate of up to 512×. As the partition point deepens, the bit-compression rate of BBNet is higher, which is the result of precision perception training. This also shows that the closer to the output point, the smaller the influence of the data in the compression layer on the final result.

**Figure 8:**
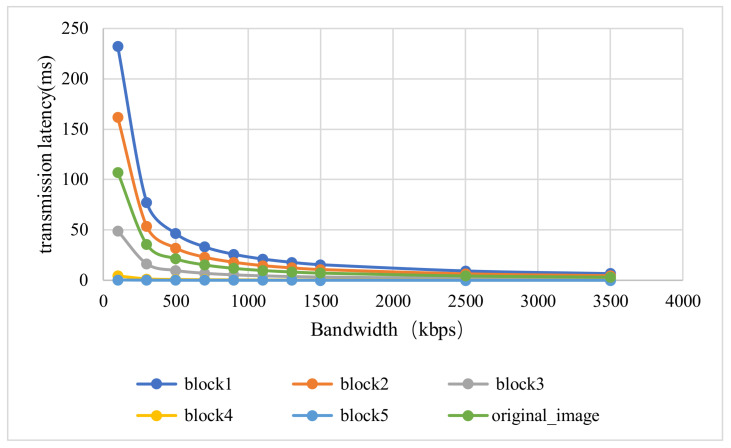
Comparison of the transmission latency of different split points of BBNet and the transmission latency of the original picture.

Different data sizes in the middle layer will inevitably affect the transmission delay. For this reason, we measured the transmission delay of different split points of BBNet under different bandwidths, as shown in Figure 8. In the experiment, to measure the accuracy, the batch size is set to 100. The transmission delay of each picture at different split points is obtained based on batch size pictures. It can be observed that as the bandwidth increases, the size of the transmitted data will have less and less impact on the transmission latency.

### 4.4. End-to-End Latency Improvement

We implemented a BBNet prototype based on NVIDIA Nano and a PC. For the ResNet18 structure without pruning strategy and feature compression layer, the accuracy tested on nano-only is 94.27%, which is called the target accuracy. Through Algorithm 1, we obtain the best split point under different bandwidth states. Moreover, the location of the best split point will change with different bandwidths. Table 4 shows the inference delays of nano-only, cloud-only, and BBNet under different bandwidths. In the experiment, the batch size is set to 100 and control the accuracy loss within 2.5%. For the end-to-end latency, if it is only divided directly between the edge and the cloud, the structure of Resnet18 cannot achieve better results than only cloud inference and edge-only inference, because no matter which layer is in the middle, the output data in the middle is much larger than the original data size.

**Figure 9:**
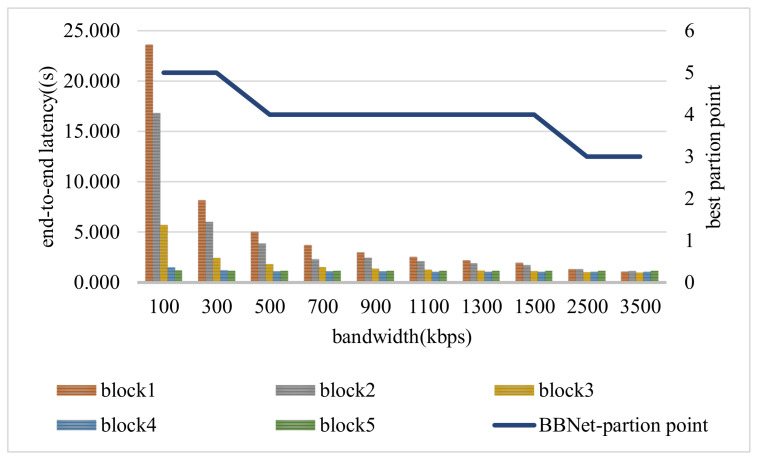
BBNet’s end-to-end latency and the change of the best split point under different bandwidths.

As shown in Figure 9, the position of the best partition point gradually moves deeper into the model as the bandwidth decreases. This shows that when the network status becomes worse, BBNet is more inclined to implement more network layers at the edge. This is because, in the case of a poor network, the communication overhead has a greater impact on the end-to-end delay than the delay executed at the edge. BBNet starts from these two aspects (the latitude and longitude of the model), so no matter how the network bandwidth changes, it can always achieve a good acceleration inference effect. For example, when the network bandwidth is the only 100kb/s, compared to cloud-only, the end-to-end delay of BBNet has achieved an acceleration effect of 9.57×. In addition, in the case of poor network conditions, the advantages of BBNet will be more obvious.

**Figure 10:**
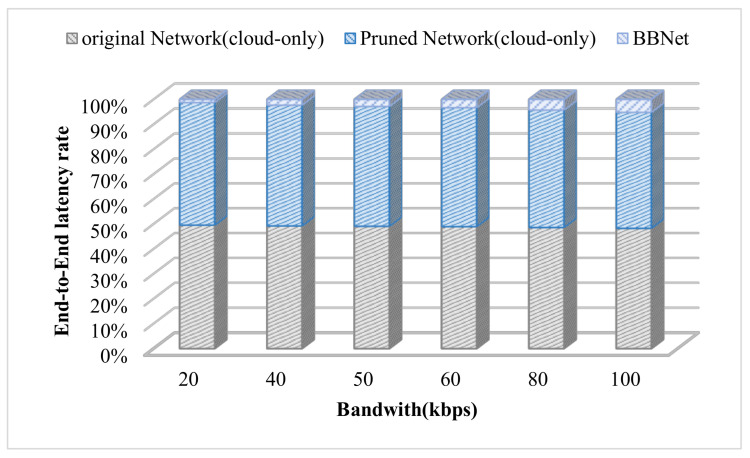
The advantage of BBNet is more obvious in the case of poor network bandwidth less than 100kbps.

From Figure 10, we can observe that when the communication environment is in extreme conditions (the communication bandwidth is less than 100kb/s), the end-to-end latency of BBNet for inference 100 pictures can still be maintained at about 1.3s. This is because in the depth of the model, the bit-compression rate become higher as less transmission delay is required. In addition, we also compared the two models for inference in the cloud (the original model and the model after pruning) and the BBNet collaborative inference method.

## 5. Summary and Future Work

This paper proposes a convolutional neural network structure for edge collaborative inference—BBNet—which combines three methods of channel-pruning, feature compression and model partition. The fusion of these three methods is rare in other works. BBNet mainly solves the problem of increased end-to-end latency after some CNN split point compared with cloud-only, because the features transmitted in the middle are larger than the size of the original data. Second, it solves the problem that the model structure cannot be directly deployed on resource-constrained edge devices. BBNet not only reduces the overall size of the model, but also accelerates edge-cloud collaborative inference in two directions (the latitude and longitude of the model). Finally, we tested the model structure in the actual hardware environment. Compared with other baselines, BBNet has lower end-to-end inference latency, and our method will perform more prominently under poor network conditions.

Although this structure has achieved good results, it still has limitations because the sparsity rate in the experiment is manually fine-tuned through experience. In addition, to find a suitable candidate model, an offline search method is used at each partition point, which will increase the training time. In future work, we will introduce reinforcement learning methods to explore the characteristics of this network structure with a large search space.

## Figures and Tables

**Table 1:** Mobile device specification.

Component	Specification
System	Jetson Nano Developer Kits
GPU	NVIDIA Maxwell w/128 NVIDIA CUDA cores
CPU	quad-core ARM Cortex-A57 64-bit
Memory	4 GB LPDDR4

**Table 2:** Cloud platform specification.

Component	Specification
System	windows 10.0
GPU	NVIDIA Geforce GTX1060 1280 NVIDIA CUDA cores
CPU	Intel(R) Core(TM) i7-8750H CPU @2.20 GHz
Memory	16 GB DDR4

**Table 3:** ResNet18 on cifar10.

	Baseline	Reduce Channel	Pruned (40%)	Fine-Tuned	Rate
Top1 accuracy $\left(\%\right)$	94.27	93.59	13.2	93.74	–
Parameters $\left(M\right)$	11.17	2.79	0.97	0.97	11.5×
FLOPs $\left(G\right)$	27.28	6.86	4.81	4.81	5.6×

**Table 4:** Comparison of different approach for CNN inference.

	Bandwidth (kbps)	Time (s)	Acc (%)	Offloaded Data (B)
nano-only	–	4.7436	94.27	–
cloud-only	100	11.378	94.27	307,200
	300	4.233	94.27	307,200
	500	2.802	94.27	307,200
	700	2.188	94.27	307,200
	900	1.850	94.27	307,200
	1100	1.635	94.27	307,200
	1300	1.486	94.27	307,200
	1500	1.376	94.27	307,200
	2500	1.093	94.27	307,200
	3500	0.970	94.27	307,200
BBNet	100	1.188	93.58	1600
	300	1.164	93.58	1600
	500	1.122	91.88	12,800
	700	1.097	91.88	12,800
	900	1.083	91.88	12,800
	1100	1.074	91.88	12,800
	1300	1.069	91.88	12,800
	1500	1.064	91.88	12,800
	2500	0.993	92.10	140,800
	3500	0.934	92.10	140,800

## Data Availability

This study analyzes publicly available datasets. This datasets can be found here: http://www.cs.toronto.edu/~kriz/cifar.html (accessed on 29 June 2021).

## Abbreviations

The following abbreviations are used in this manuscript:

DNN	Deep Neural Network
CNN	Convolutional Neural Network
ILP	Integer Linear Program
PNG	Portable Network Graphic
RB	Residual Block
